# Multi-Axis Reprogramming of Muscle–Metabolic Crosstalk by HiLo Platinum™ Restores Strength in Prediabetes via Mitochondrial Activation and Gut Microbiome Remodeling

**DOI:** 10.3390/ijms27094014

**Published:** 2026-04-30

**Authors:** Jeremy Nicolas Sibarani, Muhammad Iqhrammullah, Amal Arifi Hidayat, Ricky Indra Alfaray, Fahrul Nurkolis, Antonello Santini

**Affiliations:** 1 Faculty of Medicine, Universitas Airlangga, Surabaya 60131, Indonesia; 2 Postgraduate Program of Public Health, Universitas Muhammadiyah Aceh, Banda Aceh 23245, Indonesia; 3 Division of Gastroenterohepatology, Department of Internal Medicine, Faculty of Medicine, Universitas Airlangga, Surabaya 60131, Indonesia; 4 Helicobacter Pylori and Microbiota Study Group, Institute of Tropical Disease, Universitas Airlangga, Surabaya 60131, Indonesia; 5 Medical Research Center of Indonesia, Surabaya 60281, Indonesia; 6 Institute for Research and Community Service, State Islamic University of Sunan Kalijaga (UIN Sunan Kalijaga), Yogyakarta 55281, Indonesia; 7 Department of Pharmacy, University of Napoli Federico II, Via Domenico Montesano, 49, 80131 Napoli, Italy

**Keywords:** prediabetes, sarcopenia, whey protein, hydroxymethylbutyrate (HMB), glucosamine, muscle strength, gut microbiome, PGC-1α, SIRT-1, nutritional intervention

## Abstract

Prediabetes is increasingly recognized as a risk factor for sarcopenia, driven by chronic low-grade inflammation, insulin resistance, and impaired anabolic signaling. Nutritional interventions containing whey protein, hydroxymethylbutyrate (HMB), glucosamine, and micronutrients may offer a multi-target strategy to counteract muscle deterioration. This study aimed to evaluate the efficacy of HiLo Platinum™ supplementation in attenuating muscle strength decline in a prediabetic rat model, with integrated analysis of metabolic biomarkers and gut microbiome profiles. A randomized preclinical trial was conducted using male Sprague Dawley rats assigned to four groups: normal diet (ND), prediabetic control induced by cholesterol- and fat-enriched diet with fructose (CFEDF), and two treatment groups receiving low-dose (0.63 g/kg BW) or high-dose (1.26 g/kg BW) HiLo Platinum™. The intervention lasted six weeks. Muscle strength was assessed via a four-limb grip strength test (reverse hang time and holding impulse). Biomarkers related to inflammation, mitochondrial function, and anabolic signaling (TNF-α, IL-10, PGC-1α, IGF-1, SIRT-1, AMPK, mTOR, and myostatin), lipid profile, and blood glucose were analyzed. Gut microbiome composition and diversity were evaluated using taxonomic profiling and multivariate analyses. HiLo Platinum™ supplementation significantly improved muscle strength, evidenced by increased reverse hang time and holding impulse (*p* < 0.001). Both doses reduced blood glucose and improved lipid profiles, including increased HDL and decreased LDL, triglycerides, and total cholesterol. Anti-inflammatory effects were observed with reduced TNF-α and elevated IL-10 levels. Mitochondrial and metabolic regulators (PGC-1α, SIRT-1, AMPK) and anabolic mediators (IGF-1) were significantly upregulated, while mTOR levels decreased. Gut microbiome analysis revealed increased genus richness (Chao1 index) and distinct microbial shifts associated with improved metabolic and inflammatory markers. HiLo Platinum™ effectively mitigates prediabetes-induced muscle strength decline through integrated modulation of inflammatory pathways, mitochondrial function, metabolic homeostasis, and gut microbiome composition. These findings support its potential as a nutritional therapeutic strategy for preventing sarcopenia in prediabetic conditions, although further studies are needed to evaluate long-term effects and implications on muscle hypertrophy.

## 1. Introduction

Global prevalence of prediabetes, as defined by impaired glucose tolerance or impaired fasting glucose, has reached 9.1% and 5.8%, respectively [1]. Prediabetic conditions have been proposed as a risk factor for loss of appendicular skeletal muscle mass and sarcopenia [2,3]. Among elderly populations, specifically in males, prediabetes has been noted as an independent risk factor for sarcopenia [4]. This association may be mediated through several mechanisms, including insulin resistance, which impairs glucose uptake in skeletal muscle, leading to reduced anabolic signaling and muscle protein synthesis. Additionally, chronic low-grade inflammation and hyperglycemia in prediabetic states can exacerbate oxidative stress and promote muscle catabolism. Moreover, diabetic neuropathy, which can develop even in prediabetic stages, contributes to impaired neuromuscular function and reduced muscle strength.

Chronic inflammation in prediabetes exacerbates oxidative stress, impairing mitochondrial function and accelerating muscle degradation [5,6]. Tumor necrosis factor-alpha (TNF-α) is found at distinct levels among individuals with prediabetes as compared to type 2 diabetes mellitus (T2DM) and healthy groups [7]. As observed in a rat model, TNF-α contributes to muscle wasting by activating the tumor necrosis factor receptor 1/nuclear factor kappa-B (NF-κB) pathway, which eventually triggers the phosphorylation of insulin receptor substrate-1 (IRS-1) [8]. Additionally, TNF-α activates the ubiquitin–proteasome system, increasing muscle protein degradation [9]. Among sarcopenic individuals, along with the increase in proinflammatory cytokines, reduction of interleukin-10 (IL-10) levels is also reported [10].

Myostatin is typically elevated in insulin-resistant states, where it suppresses the PI3K/Akt/mTOR pathway, reducing protein synthesis and promoting muscle loss [11,12]. Inhibition of mammalian target of rapamycin (mTOR) diminishes anabolic signaling, leading to reduced muscle growth [12]. However, a previous study reported reduced myostatin levels in prediabetes, suggesting a possible compensatory response to early-stage muscle loss [13]. Despite this reduction, muscle wasting may still progress due to dominant catabolic factors that override the anabolic effects of lower myostatin levels. The PI3K/Akt/mTOR pathway is also influenced by insulin-like growth factor 1 (IGF-1) [14], which promotes muscle synthesis but is often diminished in prediabetic conditions, further impairing anabolic processes.

In addition to disrupted anabolic signaling, energy metabolism pathways are adversely affected in prediabetes. Reduced expression of peroxisome proliferator-activated receptor gamma coactivator 1-alpha (PGC-1α) impairs mitochondrial biogenesis and oxidative metabolism, leading to decreased energy production and muscle fatigue [15,16]. Similarly, chronic metabolic stress inhibits AMP-activated protein kinase (AMPK) activity, weakening its role in maintaining muscle mass and metabolic homeostasis. Sirtuin 1 (SIRT-1) interacts with both PGC-1α and AMPK to support mitochondrial function and insulin sensitivity [17,18].

A review article has highlighted the contributions of gut dysbiosis and glucose dysregulation in causing sarcopenia, particularly among the elderly population [19]. A study reported that *Proteobacteria* were more abundant among individuals with prediabetes than healthy controls [20]. The abundance of *Paraprevotella* was significantly lower in type 2 diabetes mellitus and prediabetes groups as compared to healthy controls [20]. An altered profile of gut microbiome may induce oxidative stress, systemic inflammation, and less production of promuscle metabolism SCFAs such as butyrate [21,22,23]. Additionally, gut dysbiosis may also affect nutrient absorption, including amino acids critical for muscle protein synthesis [24].

To manage or prevent prediabetes-induced sarcopenia, researchers have explored various nutritional intervention strategies such as the supplementation of leucine, omega-3 fatty acids, vitamin D, and probiotics. A meta-analysis also reported the effectiveness of hydroxymethylbutyrate (HMB) supplementation in maintaining muscle strength [25]. Another previously reported supplement, glucosamine, was found to possess anti-inflammatory properties that could benefit the prevention of muscle wasting [26]. It is worth noting that glucosamine is well-known for improving joint health and muscle function, particularly in metabolic disorders [26]. HiLo Platinum™ may offer a comprehensive approach to counteracting prediabetes-induced muscle weakness as it contains HMB and glucosamine, α-linolenic acid, linoleic acid, vitamin A, vitamin C, vitamin B complex, folic acid, vitamin D, and vitamin E [27]. Our previous work has found that HiLo Platinum™ targets key proteins involved in sarcopenia and Alzheimer’s disease, including angiotensin-converting enzyme (ACE), matrix metallopeptidase 9 (MMP9), and acetylcholinesterase (AChE), which are critical in regulating inflammation, muscle degradation, and neuromuscular function [27]. An in vivo investigation using a rat model was chosen, as diet-induced prediabetes in rats has been shown to significantly compromise muscle strength, as evidenced by the reverse hanging test [28].

The aim of this study was to investigate the efficacy of HiLo Platinum™ supplementation in mitigating muscle strength reduction in prediabetic male Sprague Dawley rats, with observations on biomarkers and gut microbiome profile. To our knowledge, this study is among the first to adopt an integrative, multi-axis approach to investigate prediabetes-induced muscle dysfunction by concurrently examining mitochondrial regulation, inflammatory signaling, and gut microbiome dynamics within a unified experimental framework. While previous studies have primarily focused on isolated pathways, such as anabolic signaling, inflammation, or microbiota alterations, this work provides a systems-level perspective linking these interdependent mechanisms to functional muscle outcomes. In particular, the inclusion of gut microbiome profiling alongside key regulators of mitochondrial biogenesis (PGC-1α, SIRT-1, AMPK) and inflammatory balance (TNF-α, IL-10) offers novel insight into how nutritional interventions may coordinate host–microbiome interactions to restore muscle–metabolic homeostasis. This integrative design distinguishes the present study and contributes to a more comprehensive understanding of the pathophysiology and potential therapeutic targeting of prediabetes-associated sarcopenia.

## 2. Results

### 2.1. Blood Glucose

**Figure 1:**
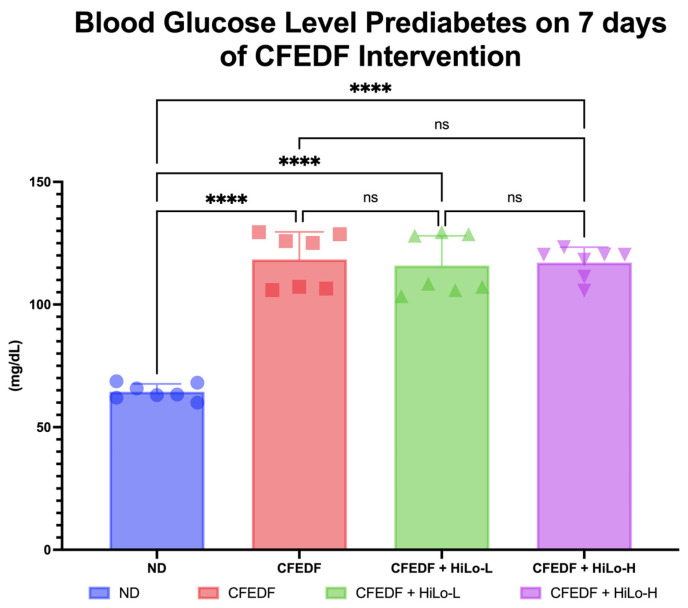
Blood glucose level was measured after prediabetes induction with the Cholesterol- and Fat-Enriched Diet with Fructose (CFEDF) for 7 consecutive days. Data are presented as mean ± standard deviation (*n* = 7). **** Statistical significance at *p* < 0.001; ns, not significant; HiLo-L, low-dose HiLo Platinum™ supplementation; HiLo-H, high-dose HiLo Platinum™ supplementation; ND, normal diet control.

**Figure 2:**
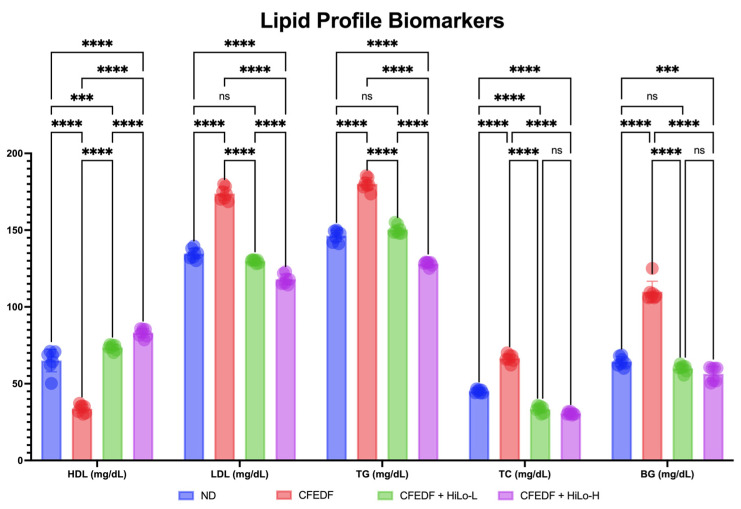
Lipid profile and blood glucose level were measured after the 6-week treatment. Data are presented as mean ± standard deviation (*n* = 7). **** Statistical significance at *p* < 0.001; *** statistical significance at *p* < 0.01; ns, not significant; HiLo-L, low-dose HiLo Platinum™ supplementation; HiLo-H, high-dose HiLo Platinum™ supplementation; ND, normal diet control.

After seven days fed with CFEDF, the blood glucose level rose significantly at *p* < 0.001 for each group when compared with ND (Figure 1). The blood glucose levels were 64.43 ± 3.22, 118.4 ± 11.21, 115.9 ± 12.14, and 117.1 ± 6.29 mg/dL for ND, CFEDF, CFEDF + HiLo-L, and CFEDF + HiLo-H, respectively. No animals were excluded due to welfare issues or death before the intervention duration ended. Following six weeks of supplementation, regardless of the supplementation dose, improvements were achieved in both supplementation groups at *p* < 0.001 (Figure 2). The blood glucose level was lower in the CFEDF + HiLo-H group than in the ND group (*p* < 0.001). There was no significant difference in blood glucose between CFEDF + HiLo-H and CFEDF + HiLo-L (*p* = 0.255). In this observation, the blood glucose remained significantly higher in the CFEDF group as compared to the ND group (*p* < 0.001, Figure 2).

### 2.2. Lipid Profile

Findings from the observation on lipid profile are presented in Figure 2. Significantly lower HDL and higher TC, LDL, and TG were observed in CFEDF as compared with ND (all with *p* < 0.001). Levels of HDL were increased after the low- and high-dose HiLo Platinum™ supplementation when compared with the CFEDF group or even the ND group (all with *p* < 0.001). The low-dose supplement significantly reduced the LDL and TG levels with *p* < 0.001, respectively. At a higher dose, LDL and TG levels were not only significantly lower than in the CFEDF group but also significantly lower than in the ND group (*p* < 0.001, respectively). Hence, the improvement in HDL, LDL, and TG in both supplementation groups was dose-dependent. As for TC, the reduction appeared in both supplementations when compared to the CFEDF (*p* < 0.001) group or the ND group (*p* < 0.001). Statistical significance was not achieved between CFEDF + HiLo-L and CFEDF + HiLo-H groups in terms of TC level (*p* = 0.481).

### 2.3. Body Weight and Behavior

**Figure 3:**
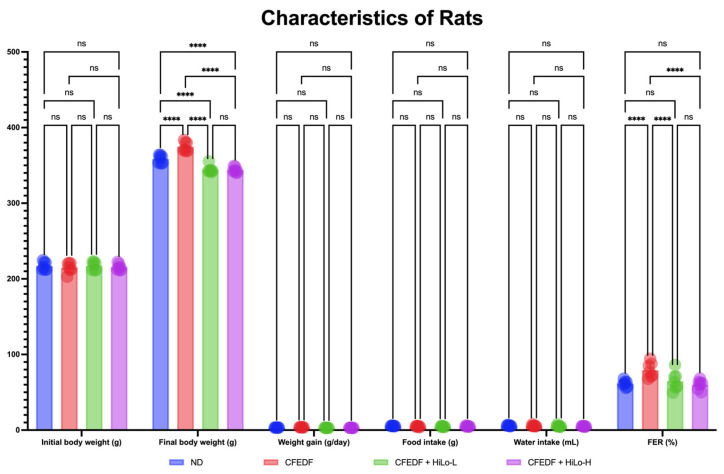
Body weight, as well as food and water intake behaviors, measured after the 6-week treatment. Data are presented as mean ± standard deviation (*n* = 7). **** Statistical significance at *p* < 0.001; ns, not significant; HiLo-L, low-dose HiLo Platinum™ supplementation; HiLo-H, high-dose HiLo Platinum™ supplementation; ND, normal diet control.

Total body weight, body weight gain, food and water intakes, and FER observed at the end of the study are presented in Figure 3. No statistical significance in initial total body weight was observed across all groups. In the CFEDF group, the total body weight was significantly higher compared with the ND group (*p* < 0.001). Following the treatment with HiLo Platinum™ supplementation, the total body weight was significantly reduced as compared to ND or CFEDF, regardless of the supplementation dose, where the *p*-values reached <0.001 for all comparisons. However, the weight gains were not significantly different across all groups. As for the food and water intakes, the behaviors were similar across all groups. FER was observed to be higher in the CFEDF group than in the ND group with *p* < 0.001. It was reduced similarly to that of the normal condition in both supplementation groups (*p* < 0.001, respectively).

### 2.4. Muscle Strength

**Figure 4:**
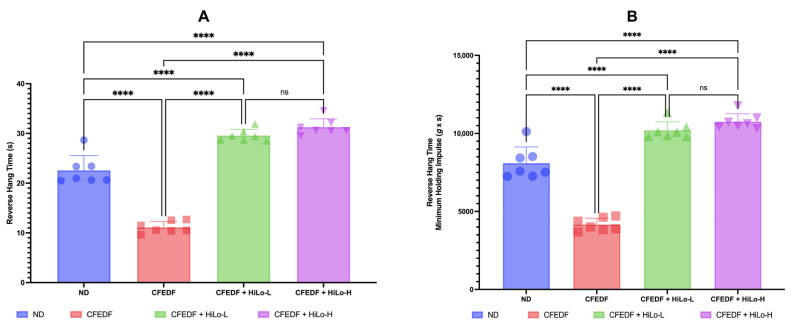
Time (**A**) and minimum holding impulse (**B**) were measured in reverse hanging tests following the 6-week treatment. Data are presented as mean ± standard deviation (*n* = 7). **** Statistical significance at *p* < 0.001; ns, not significant; HiLo-L, low-dose HiLo Platinum™ supplementation; HiLo-H, high-dose HiLo Platinum™ supplementation; ND, normal diet control.

The reverse hanging time and minimum holding impulse were significantly reduced in the CFEDF group as compared with the ND group (*p* < 0.001 for all comparisons, Figure 4). As compared to the CFEDF group, the reverse hanging time and minimum holding impulse were increased significantly in both supplementation groups (*p* < 0.001, respectively). The reverse hanging time and minimum holding impulse were even higher when compared with the ND group, with similar statistical significance at *p* < 0.001 for all comparisons (Figure 4).

### 2.5. Effects on Biomarkers

**Figure 5:**
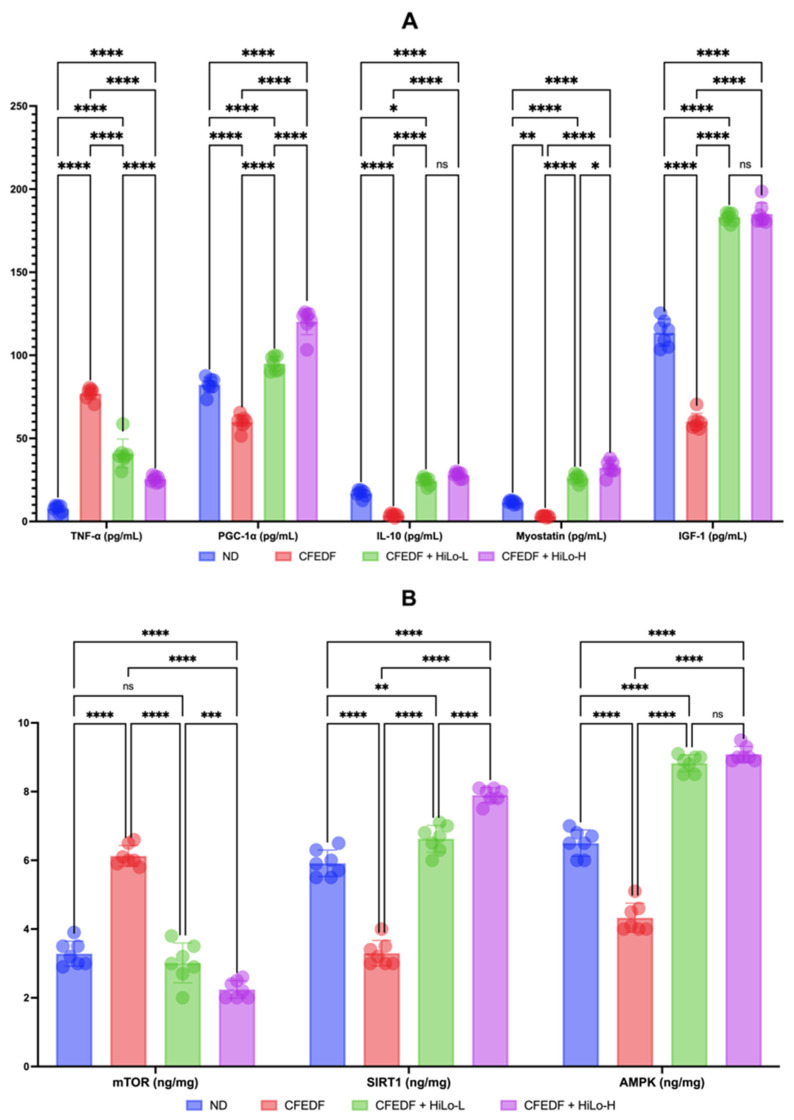
Levels of myostatin, IL-10, PGC-1α, and TNF-α (**A**). Levels of mTOR, SIRT-1, and AMPK (**B**). All biomarkers were measured after the 6-week HiLo Platinum™ supplementation. Data are presented as mean ± standard deviation (*n* = 7). **** Statistical significance at *p* < 0.001; *** statistical significance at *p* < 0.01; ** statistical significance at *p* < 0.05; * statistical significance at *p* < 0.1; ns, not significant; HiLo-L, low-dose HiLo Platinum™ supplementation; HiLo-H, high-dose HiLo Platinum™ supplementation; ND, normal diet control.

Effects of the HiLo Platinum™ supplementation on various biomarkers such as mTOR, SIRT-1, AMPK, IGF-1, myostatin, IL-10, PGC-1α, and TNF-α are presented in Figure 5. The level of TNF-α was reduced significantly by the supplementation at all doses (*p* < 0.001). However, the TNF-α level in CFEDF + HiLo-L (*p* < 0.001) and CFEDF + HiLo-H groups (*p* < 0.001) remained higher when compared with the ND group. In the case of PGC-1α, the level was significantly increased following the supplementation with low or high doses (*p* < 0.001 for all comparisons). The PGC-1α levels were higher in the supplementation groups than in the ND group (*p* < 0.001). Significant increases in IL-10 were observed in groups receiving HiLo Platinum™ supplementation (*p* < 0.001). The levels of IL-10 were higher in CFEDF + HiLo-L (*p* = 0.011) and CFEDF + HiLo-H groups (*p* < 0.001) when compared with the ND group. Myostatin levels were higher in both supplementation groups when compared with the CFEDF or ND group (all with *p* < 0.001). Similar trends of improvement were observed for IGF-1, SIRT-1, and AMPK, where the level increased following the supplementation (*p* < 0.001 for all comparisons). As for mTOR, the level was significantly reduced in supplementation groups (all with *p* < 0.001), but only the high dose gave a lower mTOR level as compared to the ND group (*p* < 0.001).

### 2.6. Effects on Liver Enzymes

**Figure 6:**
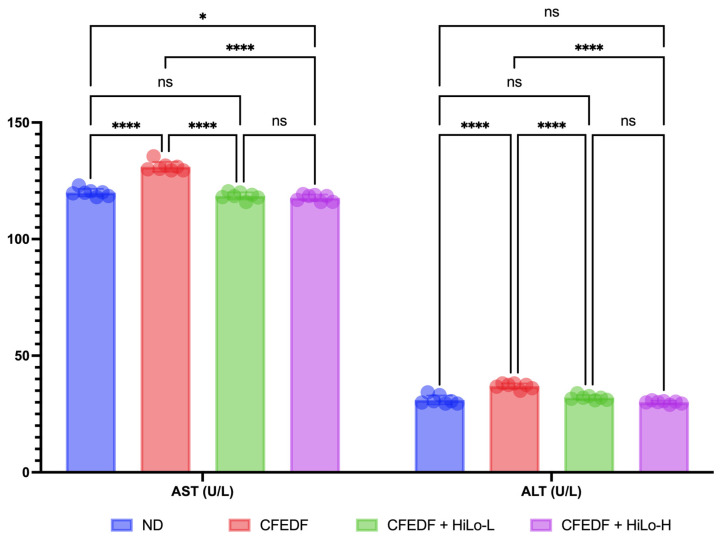
Levels of aspartate transaminase (AST) and alanine transaminase (ALT) were measured after the 6-week HiLo Platinum™ supplementation. Data are presented as mean ± standard deviation (*n* = 7). **** Statistical significance at *p* < 0.001; * statistical significance at *p* < 0.1; ns, not significant; HiLo-L, low-dose HiLo Platinum™ supplementation; HiLo-H, high-dose HiLo Platinum™ supplementation; ND, normal diet control.

The effects of HiLo Platinum™ supplementation on aspartate transaminase (AST) and alanine transaminase (ALT) are presented in Figure 6. There were significant increases in AST and ALT in the CFEDF group as compared to the ND group (*p* < 0.001). The conditions were reversed by introducing the HiLo Platinum™ supplementation regardless of the doses (*p* < 0.001 as compared with CFEDF). For AST and ALT, no significant differences were observed between the high- and low-dose groups with *p*-values of 0.728 and 0.078, respectively.

### 2.7. Effects on Gut Microbiome

#### 2.7.1. Taxonomic Composition

**Figure 7:**
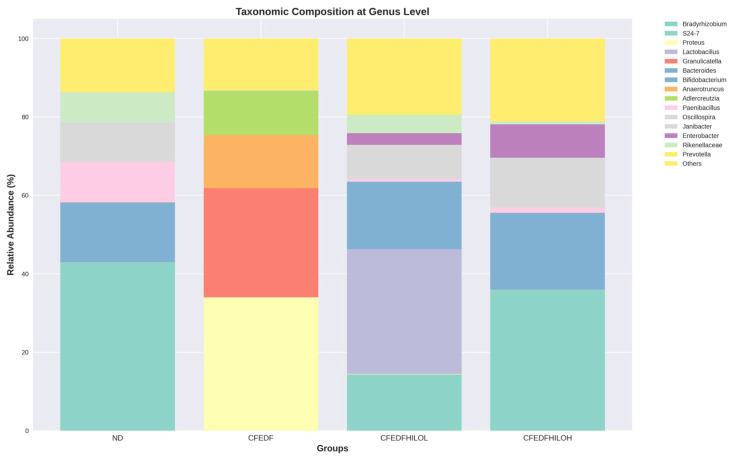
Taxonomic composition of gut microbiome assessed after the 6-week HiLo Platinum™ supplementation. Each bar represents the relative abundance of the top 15 genera, with the remaining taxa grouped under “Others”.

A graph visualizing the taxonomic composition at the genus level across ND, CFEDF, CFEDF + HiLo-L, and CFEDF + HiLo-H groups is presented in Figure 7. The top 15 predominant genera were similar among the ND, CFEDF + HiLo-L, and CFEDF + HiLo-H groups, though differences in relative abundance were still visible. Gut microbiome composition in the ND group was predominated by Bradyrhizobium, while it was no longer in the top 15 list following the CFEDF-induced prediabetes. Genus Bradyrhizobium reappeared in the top 15 list following the introduction of HiLo Platinum™ supplementation, in the CFEDF + HiLo-L and CFEDF + HiLo-H groups. As for the genera Proteus, Granulicatella, Anaerotruncus, and Adlercreutzia, they predominated in the composition only in the CFEDF group, where it was not observable among the top 15 list in other groups. Lactobacillus was uniquely abundant in CFEDF + HiLo-L.

**Figure 8:**
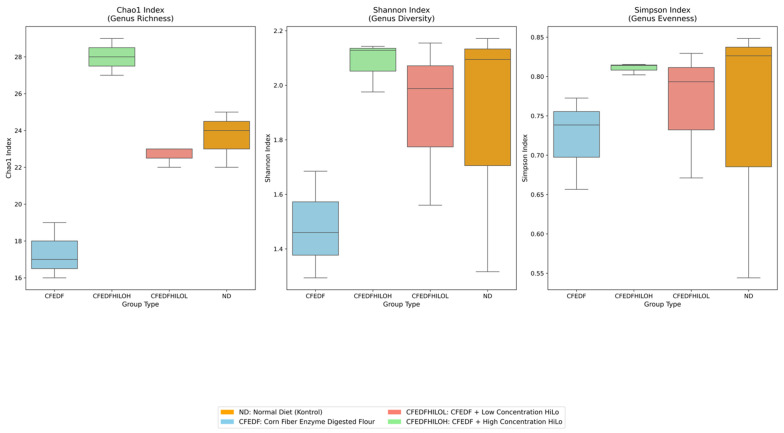
Chao1, Shannon, and Simpson indices of the gut microbiome were assessed after the 6-week HiLo Platinum™ supplementation. Data are presented as median (min–max) (*n* = 7).

Chao1 indices (genus-level richness) across all groups were found to be significantly different (H = 9.619; *p* = 0.022) (Figure 8). The highest Chao1 index was found in the CFEDF + HiLo-H group (28 ± 1), sequentially followed by ND (23.67 ± 1.53), CFEDF + HiLo-L group (22.67 ± 0.58), and CFEDF (17.33 ± 1.53). However, no statistical differences were observed in Shannon index (H = 3.923; *p* = 0.270) and Simpson index (H = 2.692; *p* = 0.442), which were the parameters for genus-level diversity and evenness, respectively. The Shannon indices, in descending order, were 2.08 ± 0.09, 1.90 ± 0.31, and 1.48 ± 0.20, corresponding to CFEDF + HiLo-H, CFEDF + HiLo-L, ND, and CFEDF, respectively. As for the Simpson index, CFEDF + HiLo-H had the highest value (0.81 ± 0.01), followed by CFEDF + HiLo-L (0.76 ± 0.08) and CFEDF (0.72 ± 0.06).

#### 2.7.2. Non-Metric Multi-Dimensional Scaling

**Figure 9:**
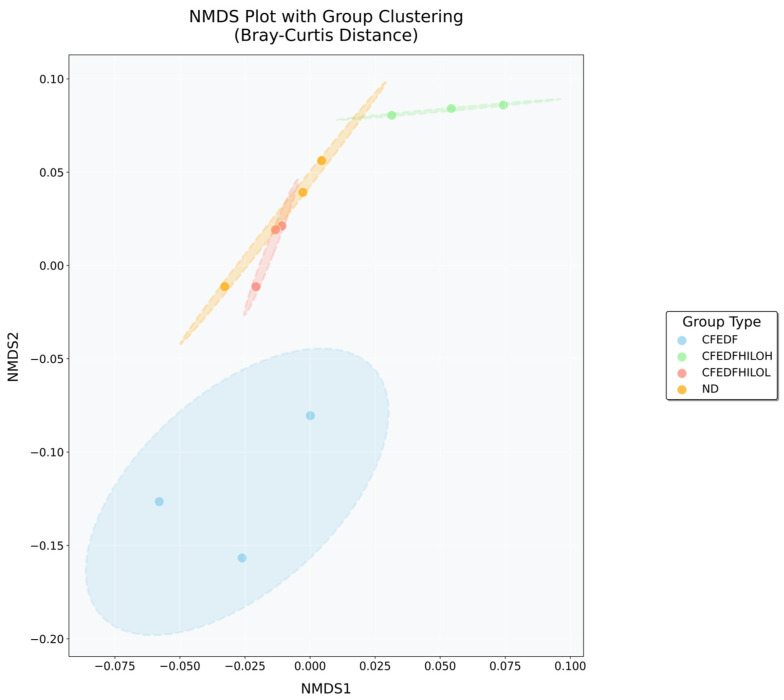
Non-metric Multi-dimensional Scaling plot based on Bray–Curtis distances for gut microbiome assessed after the 6-week HiLo Platinum™ supplementation.

Results from non-metric multi-dimensional scaling (NMDS) analysis using Bray–Curtis distances are presented in Figure 9. There were significant differences in bacterial community structure at the genus level among the treatment groups. Within-group Bray–Curtis distances were smaller than between-group distances, indicating greater consistency in community composition within each treatment group. The CFEDF + HiLo-L and CFEDF + HiLo-H groups exhibited the lowest internal variation (0.023 and 0.024, respectively), whereas the CFEDF and ND groups showed higher internal variation (0.059 and 0.054, respectively). The greatest difference in community structure was observed between the CFEDF and CFEDF + HiLo-H groups, with a Bray–Curtis distance of 0.226. In contrast, the distance between the CFEDF + HiLo-L and ND groups was relatively small (0.037). NMDS visualization revealed clear separation among the treatment groups, with 95% confidence ellipses capturing within-group variability. Both supplementation groups partially overlapped with the ND group in the ordination space, while the CFEDF group showed complete separation, positioned distinctly along the NMDS1 axis.

#### 2.7.3. Discriminative Features

**Figure 10:**
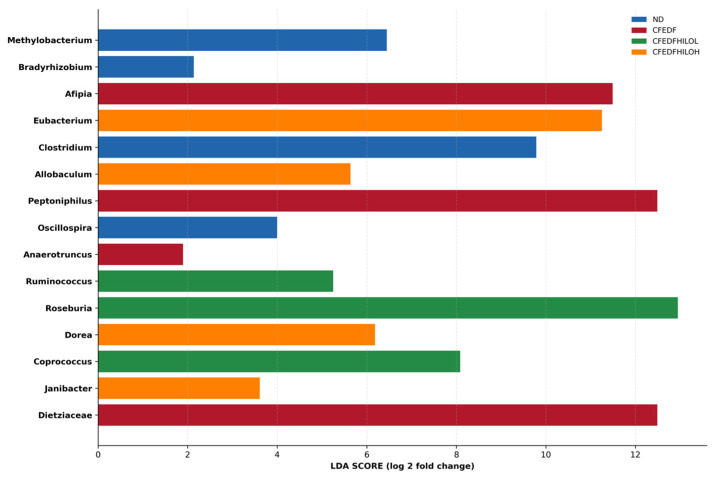
LDA scores (log-transformed) for the top 15 discriminative features of the gut microbiome assessed after the 6-week HiLo Platinum™ supplementation.

LDA scores obtained from the LEfSe analysis for the top 15 discriminative features of gut microbiomes among the animal groups were presented in Figure 10. Genera *Roseburia*, *Dietziaceae*, and *Peptoniphillus* were the three most prominent discriminative features. The compositions of *Afipia*, *Eubacterium*, and *Clostridium* were found to be discriminative according to the analysis. Other notable features included *Coprococcus*, *Dorea*, *Allobaculum*, *Methylobacterium*, and *Ruminococcus*.

#### 2.7.4. Correlation of Gut Microbiome with Biomarkers

**Figure 11:**
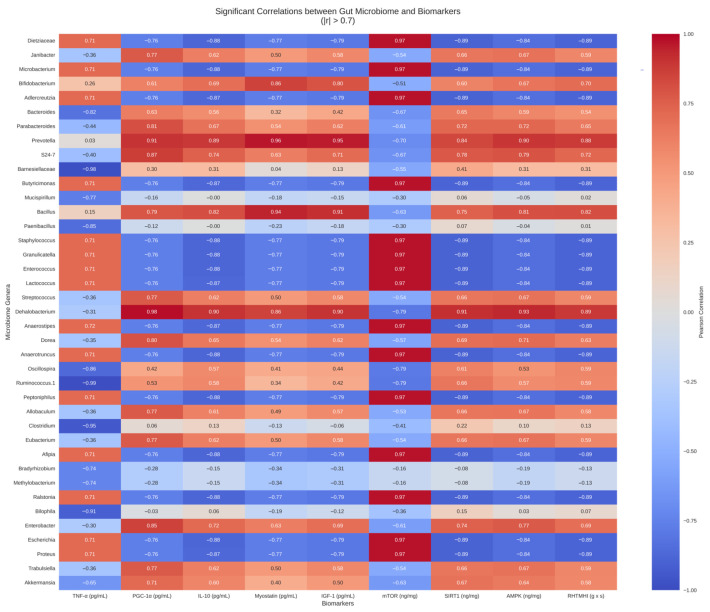
Heatmap for the correlation between gut microbiome abundance and biomarkers. Horizontal biomarker labels and a color-coded scheme distinguish positive (red) and negative (blue) correlations.

The heatmap highlights significant correlations between gut microbiome genera and biomarkers, with specific genera showing strong positive or negative relationships with biomarkers like TNF-α, PGC-1α, and mTOR (Figure 11). There were significant correlations between the abundance of the gut microbiome of a certain genus and biomarkers. Among the findings, *Dehalobacterium* had the strongest positive correlation with PGC-1α (*r* = 0.978), while *Anaerostipes*, *Lactococcus*, and *Anaerotruncus* exhibited strong positive correlations with mTOR (*r* = 0.972, respectively). *Dietziaceae* also had a positive correlation with mTOR level (*r* = 0.971). TNF-α level had the strongest correlation with *Ruminococcus* 1 (*r* = −0.985), followed by *Barnesiellaceae* (*r* = −0.978), *Clostridium* (*r* = −0.945), and *Bilophila* (*r* = −0.913). The abundance of *Anaerotruncus* was correlated with SIRT-1, with an *r*-value of −0.891 (Figure 10).

## 3. Discussions

Findings from the present study suggest that HiLo Platinum™ supplementation significantly improves skeletal muscle strength and metabolic health. Both low- and high-dose HiLo groups had higher reverse hang times and minimum holding impulses, indicating better neuromuscular coordination, endurance, and muscle power compared to prediabetic and healthy controls. HiLo Platinum™ also reduced body weight and blood glucose levels, with higher doses lowering glucose below healthy control levels, without altering food or water intake. Lipid profiles improved, with increased HDL and decreased LDL, total cholesterol, and triglycerides, the latter dropping below healthy control levels in the high-dose group. HiLo Platinum™ also enhanced genus richness, reduced inflammatory biomarkers, boosted mitochondrial biogenesis, and improved muscle function.

These findings support our previous investigation on the effect of whey protein supplement fortified with hydroxymethylbutyrate (HMB) and glucosamine in attenuating dexamethasone-induced cell death [27]. Improved muscle strength as a result of HMB supplementation has been proven in a meta-analysis of randomized controlled trials [25]. While clinical trials suggest the efficacy of glucosamine for enhancing joint health [29], previous in vivo investigations suggest that supplementation may potentially induce skeletal muscle atrophy by impairing muscle formation [30]. In a Mendelian randomization study, however, glucosamine was not associated with decreased hand grip strength [31]. On the contrary, glucosamine was found to correlate with delayed sarcopenia [31]. Other components in the whey protein drink, such as protein, calcium, α-linolenic acid, and vitamin D, collectively contribute to muscle formation, maintenance, and performance [32,33,34].

Herein, HiLo Platinum™ supplementation was found to be effective in attenuating inflammation, as observed by a reduction of TNF-α and an elevation of IL-10 levels. The findings are similar to those reported previously, where other anti-inflammatory supplements were used [35]. In a rat model study, glucosamine supplementation was reported to improve the circulating inflammatory factors [36,37]. In the present study, PGC-1α was significantly elevated in both low- and high-dose HiLo groups compared to prediabetic and healthy controls. Similarly, SIRT-1 levels were dose-dependently increased in the present study, further supporting mitochondrial health by reducing oxidative stress and improving cellular energy homeostasis. In line with a previous study, rats with T2DM exhibited a disrupted SIRT-1/PGC-1α axis, affecting mitochondrial function and increased oxidative stress [38]. During the prodromal stages of diabetes, a previous study found reduced levels of testicular PGC-1α and SIRT-3 [39].

It is worth noting that HiLo Platinum™ supplementation in the present study resulted in reduced mTOR and myostatin levels alongside increased SIRT-1, PGC-1α, and AMPK levels. While mTOR and myostatin were reduced, muscle strength still showed a significant improvement. The activation of the SIRT-1/AMPK signaling pathway likely improved mitochondrial function and energy metabolism [40]. We propose that the reduction in mTOR and myostatin may represent a compensatory mechanism to redirect the energy towards enhancing mitochondrial efficiency and oxidative metabolism, which subsequently improves muscle performance. These findings are in line with a previous study where the improvement in AMPK/SIRT-1/PGC-1α could attenuate skeletal muscle loss induced by impaired oxidative stress balance and mitochondrial function [41]. Unfortunately, constrained traditional anabolic pathways could be a disadvantage for groups like athletes or patients recovering from muscle-wasting conditions, who need to build larger muscle mass. Therefore, this trade-off between metabolic efficiency and muscle growth requires further research to better understand its implications for these specific populations.

In regard to its effect on gut microbiome, HiLo Platinum™ supplementation led to the highest genus richness (Chao1 index) in the high-dose group. However, Shannon and Simpson indices showed no significant changes, suggesting HiLo Platinum™’s effects are limited to richness rather than overall diversity or uniformity. The NMDS revealed high similarity in microbiota composition between the HiLo Platinum™-treated groups and healthy controls. The LDA score-based analysis identified distinct microbial genera associated with different groups. Prevalence of *Dietziaceae*, *Peptoniphilus*, *Afipia*, and *Anaerotruncus* characterized the prediabetic group, suggesting these genera may be linked to the inflammatory and metabolic dysregulation observed in prediabetes. Conversely, *Roseburia*, *Coprococcus*, and *Ruminococcus* were enriched in the low-dose treatment group, while *Eubacterium*, *Dorea*, *Allobaculum*, and *Janibacter* were enriched in the high-dose group.

**Figure 12:**
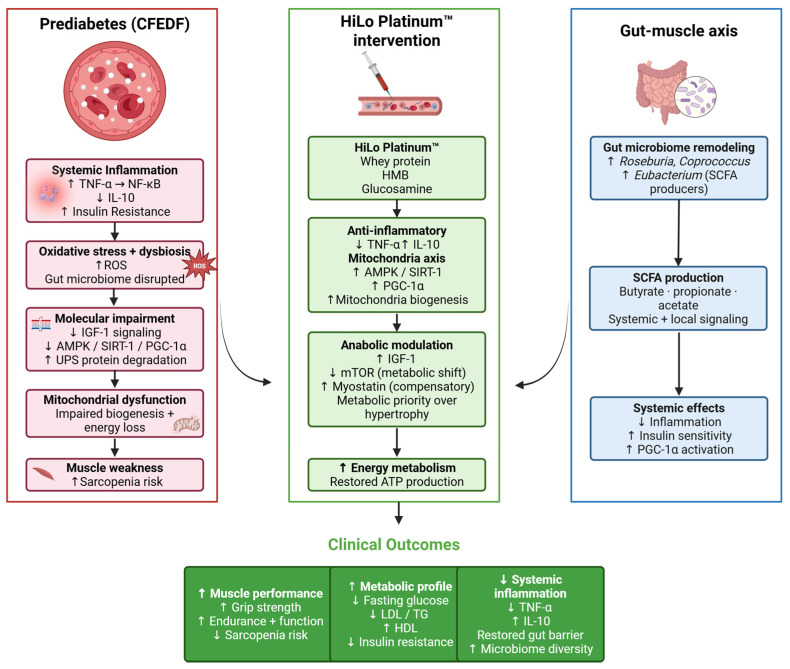
Proposed Mechanistic Pathway of HiLo Platinum™ in Mitigating Prediabetes-Induced Muscle Dysfunction. Prediabetic conditions promote inflammation, insulin resistance, and mitochondrial dysfunction, leading to impaired muscle strength. HiLo Platinum™ exerts multi-target effects by reducing TNF-α, increasing IL-10, activating the AMPK/SIRT-1/PGC-1α axis, and modulating gut microbiome composition, resulting in improved metabolic homeostasis and muscle performance. Notably, suppression of mTOR and elevation of myostatin suggest a metabolic prioritization toward mitochondrial efficiency rather than hypertrophic growth. Created in Biorender (https://biorender.com) by Fahrul Nurkolis (2026).

The strong positive correlation between PGC-1α and *Dehalobacterium* suggests a link between mitochondrial biogenesis and gut microbiota. This finding suggests that *Dehalobacterium* may contribute to enhanced oxidative metabolism in skeletal muscle, as indicated in a previous study [42]. Similarly, the correlations between mTOR and genera such as *Anaerostipes*, *Lactococcus*, and *Dietziaceae* suggest these microbes may influence anabolic pathways in muscle. Among these, *Dietziaceae* was a discriminative feature for the prediabetic group. On the other hand, TNF-α showed negative correlations with several genera, including *Ruminococcus*, *Barnesiellaceae*, and *Clostridium*, reinforcing the notion that HiLo Platinum™-mediated changes in gut microbiota composition contribute to its anti-inflammatory effects. In previous studies, some species in the aforementioned genera are attributed to both localized and systemic inflammation [43]. On the other hand, some species from *Anaerotruncus*, *Roseburia*, *Coprococcus*, and *Eubacterium* have been revealed to produce short-chain fatty acids [44,45,46,47]. For *Anaerotruncus*, there is currently no evidence for its health benefits [44], and its enrichment was found among individuals with anal fistula [48]. In the present study, *Anaerotruncus* was more predominant in the prediabetic group and was negatively correlated with SIRT-1 level. The enrichment of genera such as *Roseburia*, *Coprococcus*, and *Eubacterium* was observed in HiLo Platinum™-treated groups. The summary of the possible mechanistic pathways of HiLo Platinum™ in mitigating prediabetes was presented in Figure 12.

A key strength of the present study lies in its integrative, multi-axis approach, simultaneously examining mitochondrial regulation, inflammatory signaling, and gut microbiome dynamics within a single experimental framework. Unlike previous studies that typically focus on isolated mechanisms, this work demonstrates how HiLo Platinum™ orchestrates coordinated improvements across the AMPK/SIRT-1/PGC-1α axis, pro- and anti-inflammatory cytokines, and microbiome composition. This systems-level perspective provides a more comprehensive understanding of muscle–metabolic crosstalk in prediabetic conditions. Importantly, the integration of functional outcomes (muscle strength), biochemical biomarkers, and microbiome profiling enhances the translational relevance of the findings, supporting the concept that nutritional interventions can modulate interconnected biological networks rather than single pathways.

Despite these strengths, several limitations should be acknowledged. First, the relatively short intervention duration (six weeks) limits the ability to assess long-term sustainability, safety, and potential effects on muscle hypertrophy. Second, while the preclinical rat model provides mechanistic insights, direct translation to human physiology remains uncertain, particularly given differences in metabolism, microbiome composition, and dosing strategies. Third, although associations between microbiome shifts and host biomarkers were identified, causality cannot be established and warrants further investigation using targeted or germ-free models. Future studies should incorporate longer intervention periods, functional muscle mass assessments, and clinical validation to determine the applicability of these findings in human populations, particularly in individuals at risk of sarcopenia or metabolic disorders.

## 4. Materials and Methods

### 4.1. Study Design

The primary aim of this study was to assess the effects of HiLo Platinum™ supplementation on the muscle strength of prediabetic male Sprague Dawley rats based on the four-limb grip strength test. To unveil the mechanisms, we further assessed the myostatin, insulin-like growth factor 1 (IGF-1), Akt/mTOR pathway proteins, PGC-1α, TNF-α, IL-10, lipid profile, AST, and ALT levels. In addition, we observed the changes in body weight and food and water intakes following the supplementation. A special feed formulation, cholesterol- and fat-enriched diet with fructose (CFEDF), was prepared to induce the prediabetic condition. For blinding procedures, intervention providers, diet providers, and data analysts were not informed about the type of diet or feed given to the groups. Outcome assessment was also conducted under blinding to ensure unbiased evaluation. All experimental procedures involving animals were conducted in accordance with the Guidelines for Reporting In Vivo Experiments (ARRIVE). The study protocol was registered and approved in the Preclinical Trials Study Register (registration number: PCTE0000566). Ethical approval was also obtained from the Institute for Research and Community Services, Sunan Kalijaga State Islamic University, Yogyakarta (Approval No: 2390.2/Un. 02/L3/TL/06/2025).

### 4.2. Animal and Randomization

We used healthy male Sprague Dawley rats aged 3–5 weeks, obtained from the Animal Husbandry Farm, Indonesia. The minimum sample size was estimated by Federer’s formula ((number of groups − 1) × (minimum number of rats − 1) ≥ 15) [49,50]. A minimum sample size of 7 rats per group was chosen to avoid animal dropouts. Upon arrival, the rats were acclimatized to laboratory conditions for 7 days before the experiment. All rats were provided free access to food and water ad libitum and housed in cages under standard laboratory conditions (temperature: 27 ± 2 °C) with a 12/12 h light–dark cycle. Professional vets observed the animals daily for signs of welfare issues (such as lack of appetite, ruffling, lethargy, indifference, hiding, or curling up) and examined them weekly for health and weight changes. Rats were randomly assigned to four groups, namely normal diet control (ND), negative control (CFEDF), prediabetic rats supplemented with low-dose HiLo Platinum™ (HiLo Platinum™-L), and prediabetic rats supplemented with high-dose HiLo Platinum™ (HiLo Platinum™-H). The group assignment was carried out in random order to one of the four groups (ND, CFEDF, CFEDF + HiLo-L, CFEDF + HiLo-H) until all rats were assigned. The ND group received a standard pellet diet and ad libitum water. The CFEDF group received a CFEDF and ad libitum water. The CFEDF + HiLo-L group received 0.63 g/kg BW HiLo Platinum™, a CFEDF, and ad libitum water. The CFEDF + HiLo-H group received 1.26 g/kg BW HiLo Platinum™, a CFEDF, and ad libitum water.

### 4.3. Prediabetic Induction and Treatment

Administration of CFEDF induced rats to become prediabetic. A dose of HiLo Platinum™ was administered orally by a trained professional. During the whole experiment, the daily intake of animal feed and drinking fluids was monitored so it did not differ between the control and experimental groups. After six weeks of rats’ interventional feeding, a blood sample was extracted. Preparation included rats fasting the night before blood was drawn, with ketamine used as anesthesia. The venous sinus was the chosen location to draw blood; the collected blood was then put in a sterile and dry tube with no anticoagulant and was allowed to coagulate at room temperature. Then, centrifugation (3000 rpm, 20 min) was done to obtain the serum.

Cholesterol- and fat-enriched diet with fructose (CFEDF) is a standard rat diet containing 1% cholic acid, 2% pure cholesterol powder, 20% fat (animal source/pork oil), and 2% corn oil and supplemented with 15% fructose-enriched water. All additional components were added smoothly into the standard CFEDF, mixed thoroughly until homogeneous, formed into a dough by adding 1000 mL of distilled water, rolled using a bottle, cut into small pellets, and left to dry at room temperature under sterile conditions. CFEDFs were prepared weekly and stored at 4 °C until use to reduce oxidation of CFEDFs by air.

### 4.4. Four-Limb Grip Strength Test

The four-limb grip strength test was conducted at the end of the treatment duration to evaluate neuromuscular function, specifically measuring reverse hang time and minimum holding impulse. A wire mesh grid with dimensions of 80 × 50 cm, wire mesh spacing of 12 mm, and wire thickness of 1.5 mm was used for the test. The grid was positioned at a height of approximately 60 cm, with soft bedding placed underneath to protect the rats in case of a fall. Each rat was gently placed in a face-up position, allowing it to grasp the mesh grid with all four limbs. The grip-holding time was measured until the rat released its grip. The procedure was repeated twice for each rat by the same assessor to ensure consistency, and the longest reverse hang time was recorded. The rat was given an interval of 60 s for resting before the next repetition. To account for the influence of body mass on grip performance, the minimum holding impulse was calculated using the following formula: body mass (g) × time (s).

### 4.5. Endpoints

The primary endpoint of this study was neuromuscular performance, assessed using the four-limb grip strength test. The measured parameters included reverse hang time, defined as the duration the rat was able to maintain its grip while inverted, and minimum holding impulse, calculated as body mass (g) multiplied by hang time (s), reflecting muscle endurance and strength. These outcomes were selected to provide complementary functional indicators of skeletal muscle performance following HiLo Platinum™ supplementation.

### 4.6. Collection and Preparation of Tissue and Blood for Biomarker Assessment

Blood glucose and cholesterol levels were tested using a COBAS Integra^®^ 400 plus analyzer (Roche Diagnostics, Mannheim, Germany). Blood samples from liver tissue were washed with 1% phosphate buffered saline (PBS, pH 7.4) until the washing fluid was clear. PBS 1% of the sample at 3000 rpm was centrifuged for 20 min to obtain the pellet and supernatant. The supernatant was taken for PGC-1α examination. PGC-1α concentration was measured using a PGC Mouse 1α ELISA Kit (Sunlong Biotech Co., Ltd, Hangzhou, China). Biomarkers assessed in experimental animals include: lipid profile: HDL, LDL, TG, TC, BG; enzymatic: α-amylase, alpha-glucosidase, lipase, HMG-CoA reductase; leptin; KCNJ11 and TCF7L2 expressions; IL-10, TNFα, and PGC-1α, referring to the analysis protocol in previous studies.

### 4.7. Gut Microbiome Assessment

Fecal samples were collected aseptically at the end of the intervention period and immediately stored at −80 °C until further analysis to preserve microbial integrity. Microbial genomic DNA was extracted using a commercially available stool DNA extraction kit (OMG Soil DNA Extraction Kit, Shanghai Meiji Biopharmaceutical Technology Co., Ltd., Shanghai, China) according to the manufacturer’s protocol.

The hypervariable V3–V4 regions of the bacterial 16S rRNA gene were amplified using polymerase chain reaction (PCR) with universal primers 338F (5′-ACTCCTACGGGAGGCAGCAG-3′) and 806R (5′-GGACTACHVGGGTWTCTAAT-3′). PCR amplification was performed under optimized cycling conditions, and the resulting amplicons were purified and quantified prior to sequencing. High-throughput sequencing was conducted using the Illumina MiSeq PE300 platform, generating paired-end reads for downstream microbiome profiling.

Raw sequencing data were subjected to quality control and preprocessing using FAST software (version 3.5.2), followed by sequence assembly and trimming using FLASH software (version 1.2.11). High-quality reads were clustered into operational taxonomic units (OTUs) at a 97% similarity threshold using UPARSE software (version 7.1). Chimeric sequences were identified and removed using reference-based and de novo approaches. Taxonomic assignment was performed by aligning representative sequences against the Silva 16S rRNA database (version 138) with a minimum confidence threshold of 70%.

Microbial diversity analyses were conducted at the genus level. Alpha diversity metrics, including the Chao1 index (richness), Shannon index (diversity), and Simpson index (evenness), were calculated to evaluate within-sample microbial diversity. Beta diversity was assessed using Bray–Curtis dissimilarity and visualized through non-metric multi-dimensional scaling (NMDS) to determine differences in microbial community structure among experimental groups.

To identify discriminative microbial taxa associated with each treatment group, linear discriminant analysis effect size (LEfSe) was applied, and taxa with significant differences were reported based on LDA scores. Furthermore, correlation analysis between microbial genera abundance and metabolic or inflammatory biomarkers (e.g., TNF-α, PGC-1α, mTOR, SIRT-1) was performed using Spearman correlation coefficients, and the results were visualized as a heatmap to explore microbiome–host interaction patterns.

### 4.8. Data Analysis

Statistical analyses were performed using GraphPad Prism (version 11.0.0, GraphPad Software, San Diego, CA, USA). Data were first tested for normality using the Shapiro–Wilk test to determine the appropriate statistical tests. For normally distributed data, comparisons between groups were analyzed using one-way analysis of variance (ANOVA) followed by a Tukey post hoc test for pairwise comparisons. For non-normally distributed data, the Kruskal–Wallis test was used, followed by Dunn’s post hoc test. Results were expressed as mean ± standard deviation (SD) for parametric data or median with interquartile range (IQR) for non-parametric data. Statistical significance was set at *p* < 0.05.

## 5. Conclusions

HiLo Platinum™ supplementation effectively mitigates muscle strength reduction in prediabetic male Sprague Dawley rats. The supplementation led to significant improvements in muscle performance, as evidenced by increased reverse hang time and holding impulse. Additionally, HiLo Platinum™ positively influenced metabolic biomarkers by reducing TNF-α, while increasing PGC-1α, IL-10, IGF-1, and SIRT-1. Lipid profiles improved with higher HDL and reduced LDL, total cholesterol, triglycerides, and blood glucose levels. The gut microbiome composition showed increased genus richness and beneficial microbial shifts correlating with improved inflammatory and metabolic markers. Despite these benefits, the supplementation appeared to restrict traditional anabolic pathways, which could hinder muscle growth. This limitation may not be ideal for athletes or individuals recovering from muscle-wasting conditions who require greater muscle mass. Further research is needed to explore this balance between enhanced metabolic health and reduced muscle hypertrophy to determine its suitability for these specific groups. HiLo Platinum™ shows potential as a therapeutic strategy for preventing or managing prediabetes-induced sarcopenia, though its application may need to be tailored based on individual muscle growth requirements.

## Data Availability

The original contributions presented in this study are included in the article. The datasets used and/or analyzed during the current study are available from the corresponding authors on reasonable request. Further inquiries can be directed to the corresponding authors.
